# The promoter of *Bmlp3* gene can direct fat body-specific expression in the transgenic silkworm, *Bombyx mori*

**DOI:** 10.1007/s11248-013-9705-8

**Published:** 2013-03-30

**Authors:** DangJun Deng, HanFu Xu, Feng Wang, Xiaoli Duan, SanYuan Ma, ZhongHuai Xiang, QingYou Xia

**Affiliations:** 1 State Key Laboratory of Silkworm Genome Biology, Southwest University, Chongqing, 400715 People’s Republic of China; 2 Institute of Forensic Science, Chongqing Public Security Bureau, Chongqing, 401147 People’s Republic of China

**Keywords:** *Bmlp3* promoter, Fat body, Specific expression, Transgene, Silkworm

## Abstract

The fat body plays multiple, crucial roles in the life of silkworms. Targeted expression of transgenes in the fat body of the silkworm, *Bombyx mori*, is important not only for clarifying the function of endogenous genes expressed in this tissue, but also for producing valuable recombinant proteins. However, fat body-specific gene expression remains difficult due to a lack of suitable tissue-specific promoters. Here we report the isolation of the fat body-specific promoter of *Bmlp3*, a member of the 30K protein family of silkworms. The 1.1 kb fragment from −374 to +738 of *Bmlp3* displayed strong promoter activity in the cell lines BmE and Spli-221. In transgenic silkworms, a *DsRed* reporter gene controlled by the 1.1 kb *Bmlp3* promoter fragment was expressed specifically in the fat body in a stage-specific pattern that was nearly identical to the endogenous *Bmlp3* gene. We conclude that the 1.1 kb *Bmlp3* promoter fragment is sufficient to direct tissue- and stage-specific expression of transgenes in the fat body of silkworms, highlighting the potential use of this promoter for both functional genomics research and biotechnology applications.

**Electronic supplementary material:**

The online version of this article (doi:10.1007/s11248-013-9705-8) contains supplementary material, which is available to authorized users.

## Introduction

The fat body of the silkworm, *Bombyx mori*, is a relatively large tissue distributed throughout the body. As the central storage tissue for nutrients and energy reserves, the fat body plays crucial roles in the life of silkworms including development, metamorphosis, immunity and reproduction. Large amounts of storage proteins, lipoproteins and vitellogenins are synthesized in the fat body, many of which are released and accumulated in a sex- and stage-specific manner in the hemolymph. Consequently, the fat body represents an ideal model tissue for studying metamorphosis and the developmental regulation of gene expression (Izumi et al. 1981; Sakurai et al. 1988a, b; Sakai et al. 1988). Recently, the analysis of whole-genome DNA sequence data, large-scale expressed sequence tag data and microarray-based gene expression profiles revealed numerous tissue-specific genes responsible for the accurate regulation and prodigious biosynthesis of proteins in the fat body of *B. mori* (Xia et al. 2004, 2007; Cheng et al. 2006). These findings provide a unique opportunity to better understanding the elusive biological roles of the fat body by clarifying the function of these genes.

Analyzing gene function is greatly aided by transgenic technology in which gene expression is regulated temporally and spatially. Since the first successful germ-line transformation of *B. mori* using the *piggyBac* transposon (Tamura et al. 2000), dozens of genes have been studied using transgenic methods alone or in combination with other genetic tools. In many cases, as reported in other model organisms, the use of tissue- and/or stage-specific promoters has proven to be very important. Unfortunately, it has been difficult to study the fat body of the silkworm using this strategy because of the lack of suitably specific promoters. Moreover, considering the extraordinary ability of the fat body of the silkworm to synthesize and store large amounts of proteins, this tissue might be developed into a novel bioreactor to produce valuable recombinant proteins. To test these ideas it is necessary to isolate fat body-specific promoters that lead to high levels of gene expression in useful temporal and spatial patterns.

Among the major proteins synthesized by the fat body of *B. mori* are a group of low molecular weight lipoproteins (Bmlps) with molecular weights around 30 kDa, and known as “30K proteins”. 30K proteins, are synthesized in a specific temporal patterns and released into the hemolymph during the post-embryonic development (Tojo et al. 1980; Izumi et al. 1981,1984; Tomino 1985). So far, a total of 24 genes encoding typical 30K proteins (*Bmlp1*–*24*) have been identified in the genome of *B. mori* (Zhang et al. 2012a, b). Interestingly, at least six of them, including *Bmlp1*–*4*, *Bmlp7* and *Bmlp9*, were abundantly expressed in the fat body in a developmentally regulated pattern (Sun et al. 2007; Hou et al. 2007; Zhang et al. 2012a, b), suggesting that the regulatory regions of these genes might be useful for controlling the expression of transgenes in the fat body of silkworms.

The objective of this study was to isolate the 5′ regulatory region of *Bmlp3* and to test whether it can direct fat body-specific expression of transgenes in the silkworm. We isolated the 5′ regulatory region of *Bmlp3* and tested its activity in insect cell lines using a Dual-luciferase reporter assay, and transgenic silkworms by fusing a *Bmlp3* promoter-containing fragment to a *DsRed* reporter gene. The results showed tissue- and stage-specific expression of *DsRed* in the fat body of transgenic silkworms, demonstrating the utility of the *Bmlp3* promoter for both functional genomic studies and biotechnology applications.

## Materials and methods

### Silkworm strain

The silkworm strain *P50* was maintained in our laboratory and used for promoter isolation and germline transformation. Eggs were maintained at 25 °C with 95 % humidity until hatching, and larvae were reared on fresh mulberry leaves at 25 °C.

### Vector construction

The 1,119 bp promoter region (from −374 to +745) upstream of the *Bmlp3* gene was amplified using the primers Bmlp3-PF1/Bmlp3-PR1 (Table S1) and cloned into pMD19-T simple vector (TaKaRa). To investigate the activity of this promoter, the DNA fragment was cut with *Sma*I and *Xho*I and inserted into the pGL3-basic vector (Promega) which contained the *luciferase* reporter gene, to generate the transient expression vector Bmlp3-pGL3.

To construct the transgenic vector pBacBmlp3-*DsRed*/3 × P3EGFP, the *Bmlp3* promoter region was amplified from the plasmid Bmlp3-pGL3 using the primers Bmlp3-PF2/Bmlp3-PR2 (Table S1), and assembled in the shuttle vector pSLfa1180fa (Horn and Wimmer 2000) by fusing in turn with *DsRed* and SV40 polyA signal. Then the Bmlp3-DsRed-SV40 cassette was cloned into the unique *Asc*I site of the *piggyBac*-containing vector pBac[3 × P3EGFPafm] (Horn and Wimmer 2000) to generate the final donor vector.

### Cell culture and dual-luciferase reporter assay

The *B. mori* embryonic cell line BmE and ovarian cell line BmN, the *Spodoptera frugiperda* ovarian cell lines Sf9 and Sf21, and the *Spodoptera litura* embryonic cell line Spli-221, were maintained at 27 °C in Grace’s media supplemented with 10 % fetal bovine serum (FBS, Hyclone, China), 50 U/mL penicillin and 50 mg/mL streptomycin. For transfections, cells were seeded onto a 24-well tissue culture plate (1 × 10^5^ cells/well) for 12 h. One hundred microliters of a mixture containing 1 μg Bmlp3-pGL3 plasmid DNA, 0.1 μg pRL-SV40 plasmid DNA 3 μl LipofectAMINE 2000 (Invitrogen) was incubated in the serum-free Grace’s media with BmE, BmN, Sf9, Sf21 or Spli-221 cells for 4–6 h. Plasmid pRL-SV40 (Promega) is a 3.7 kb plasmid with the *luciferase* gene from *Renilla* *reniformis* under the regulatory control of the SV40 promoter. The transfection mixture was replaced with 500 μL fresh medium containing 10 % FBS and incubated for an additional 24 h. The transfected cells were washed twice with 1× phosphate-buffered saline (PBS) and lysed by 100 μL/well lysis Buffer (Promega), the supernatant of cell lysis were further collected by centrifugating at 12,000 rpm, under 4 °C for 15 min. Dual-Luciferase reporter assays were performed on Modulus™ single tube multimode reader (Promega) according to the manufacturer’s protocols. Luciferase activity from expression of Bmlp3-pGL3 was normalized to luciferase activity from pRL-SV40. All experiments were repeated three times independently and the results reported as mean ± SE.

### Germline transformation and image analysis

Transgenic *B. mori* were created using previously described methods (Tamura et al. 2000). Plasmids pBacBmlp3-*DsRed*/3 × P3EGFP and pHA3PIG (a *piggyBac* transposase-expressing ‘helper’ plasmid) were purified using QIAGEN Plasmid Midi Kit (Qiagen). Purified plasmid DNA was dissolved in super-pure water. The mixture of the donor and helper DNA, 400 and 400 μg/μL, respectively, was injected into the pre-blastoderm eggs 1–2 h after oviposition. G0 moths developing from injected embryos were mated with each other to generate G1 progeny. Day-7 G1 embryos were screened for EGFP expression in the ocelli and compound eyes using an Olympus MVX10 fluorescent stereomicroscope (Olympus, Japan) equipped with appropriate filters for the detection of EGFP and RFP fluorescence. EGFP-positive individuals were reared to adults and siblings were mated to generate G2 offspring. *DsRed* expression in the dissected fat bodies of G2 larva were observed using a fluorescent stereomicroscope as described above.

### Molecular analysis

#### Southern blotting

Genomic DNA from transgenic and wild-type moths was extracted using an improved phenol/chloroform method (Zhao et al. 2010). DNA samples (10 μg) were fully digested using the restriction endonucleases *Hind*III and *Xho*I. The resulting DNA fragments were size fractionasted by gel electrophoresis in 1 % (w/v) agarose. Size-fractionated DNA was stained in-gel with ethidium bromide, or transferred directly to nylon membranes (Roche Applied Science) using vacuum transfer methods. DNA-containing nylon membranes were hybridized with a digoxigenin-labeled probe specific for the EGFP open reading frame (Table S1) at 65 °C. Hybridized digoxigenin-labeled probes were detected using chemiluminescent methods with CDPStar (Roche, USA) according to the manufacturer’s instructions.

#### Inverse PCR

Inverse PCR experiments were performed to identify the genomic location of integrated *piggyBac* vectors using genomic DNA purified from transgenic G1 moths and primers (Table S1) specific to the 5′ and 3′ ends of the *piggyBac* transposon as described (Tamura et al. 2000). The inverse PCR products were cloned into the vector pMD19-T (TaKaRa) and their DNA sequences determined.

#### RT-PCR

Total RNA from transgenic and wild-type larvae was isolated using the TRIzol reagent (Invitrogen). Isolated RNA (2 mg) was treated with *DNase*I to remove trace amounts of genomic DNA and then used as templates for the synthesis of DNA. cDNA was synthesized using the Reverse Transcriptase M-MLV Kit (TaKaRa) according to the manufacturer’s protocol. The PCR reactions were performed as follows using gene-specific primers (Table S1): denaturation at 94 °C for 3 min, 26 cycles of 94 °C for 30 s, 55 °C for 30 s and 72 °C for 45 s. The PCR products were separated on 1 % agarose gels and visualized by staining with ethidium bromide.

#### Western blotting

Proteins from transgenic and wild-type day-7 fifth instar larvae were extracted with 1× PBS, incubation at room temperature overnight followed by centrifugation at 10,000 rpm in a microcentrifuge for 5 min. Samples of the supernatants were used to estimate the protein concentration by the Bradford protein assay and part was stored at −20 °C. Each sample of 10 μg protein was subjected to SDS-PAGE and transferred to a poly (vinylidene difluoride) membrane. RFP was detected using an anti-RFP antibody (Gene. Co) and His-tagged RFP (BaiRui. Co) was used as a positive control. Quantity of RFP is determined by densitometric measurement of the immunoblot using a free Quantity One software(BioRad).

## Results and discussion

### Isolation and activity detection of the *Bmlp3* promoter

**Fig. 1:**
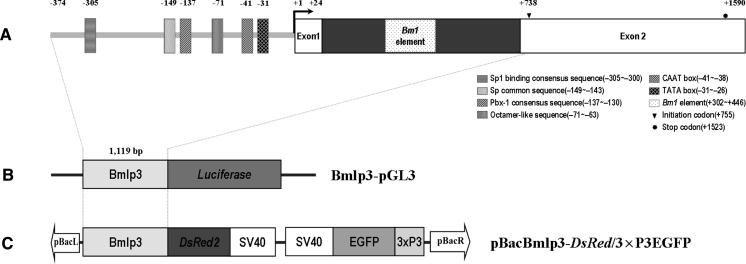
Schematic diagram of the *Bmlp3* promoter and two vectors. **a** Genomic organization of *Bmlp3*. The predicted transcription start site is denoted +1. Exons are shown as white *rectangles* and intron is shown as *black rectangles*. The initiation codon (ATG) and stop codon (TAA) are indicated by a *triangle* and *star* respectively in exon2. The TATA box, CAAT box, and various putative motifs are shown as differently *shaded boxes* as indicated in the figure. Numbers above the exon and cis-element boxes denotes the position of nucleotides in the gene relative to the transcription start site. **b** Schematic diagram of the transient expression vector Bmlp3-pGL3. **c** Schematic diagram of the transgenic expression vector pBacBmlp3-*DsRed*/3 × P3EGFP

Basing on bioinformatic analyses, a 1,119 bp genomic DNA fragment upstream of the *Bmlp3* gene was identified as putatively containing the promoter and isolated by PCR from the silkworm strain *P50*. The fragment comprised 24 bp of exon1, 713 bp of intron1, 8 bp of partial sequence of exon2 (9 bp distance from the initiation codon), and 374 bp of 5′-flanking region upstream of transcription initiation site. Besides the core promoter sequences including the TATA-box and CAAT-box, several conserved motifs reported previously were detected including Spl-binding and Pbx-1 consensus sequences, an octamer-like sequence, a *Bm1* element and a common sequence found in storage protein genes (Mori et al. 1991a, b; Sakurai et al. 1988a, b; Willott et al. 1989; Matsumoto et al. 1986; Delaney et al. 1986; Fujiwara and Yamashita 1992; Ogawa et al. 2005) (Fig. 1a).

**Fig. 2:**
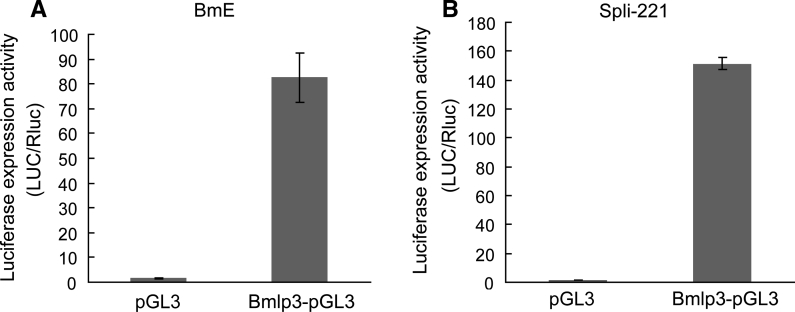
Promoter activity of the 1,119 bp fragment from the 5′ region of *Bmlp3* in insect cell lines. Expression of luciferase derived from Bmlp3-pGL3 in BmE (**a**) and Spli-221(**b**) cell lines. The activity of luciferase was measured at 72 h after co-transfection with Bmlp3-pGL3 and pRL-SV40. The results were calculated from three independent experiments and described as the mean ratio of Firefly luciferase (from Bmlp3-pGL3) to *Renilla* luciferase (from pRL-SV40) ± standard deviation

To examine the activity of the *Bmlp3* promoter, a quantitative assessment of luciferase activity was measured in five insect cell lines, BmE, BmN, Sf9, Sf21 and Spli-221, using a Dual-Luciferase reporter assay. The results showed that the *Bmlp3* promoter had highly activity in BmE and Spli-221 cells, with the activity in Spli-221 was higher than in BmE (Figs. 1b, 2). However, no activity was detected in BmN, Sf9 and Sf21 cells (data not shown), and the reason might be the lack of essential regulatory factors responsible for the activity of *Bmlp3* promoter in these cell lines. Taken together, these results suggest that the 1,119 bp fragment from the 5′ region of *Bmlp3* has promoter activity under some conditions and might be sufficient to direct expression of transgenes in the fat body of silkworm.

### Generation of the transgenic silkworm

To test whether the putative 1,119 bp *Bmlp3* promoter-containing fragment can direct fat body-specific expression in silkworms, we fused the fragment to a *DsRed* reporter gene (Fig. 1c) and then introduced the construct into the genome of *B. mori*. From 357 injected silkworm embryos a total of 126 moths were recovered and crossed, leading to 5 broods that produced EGFP-positive progeny. Transgenic G1 moths were randomly selected and genomic DNA was isolated and used to check for the presence of integrated transgene-containing *piggyBac* vectors by Southern blotting and inverse PCR analysis using an EGFP-specific probe and *piggyBac*-specific primers, respectively. The results indicated that the transgenic silkworms harbored single insertion located in Chromosome 20 (Fig. S1), suggesting only one transposition event during germ-line transformation. This transgenic line harboring single copy of the *DsRed* reporter gene was used for subsequent expression analyses.

### Fluorescent detection of *DsRed* expression in transgenic silkworm

**Fig. 3:**
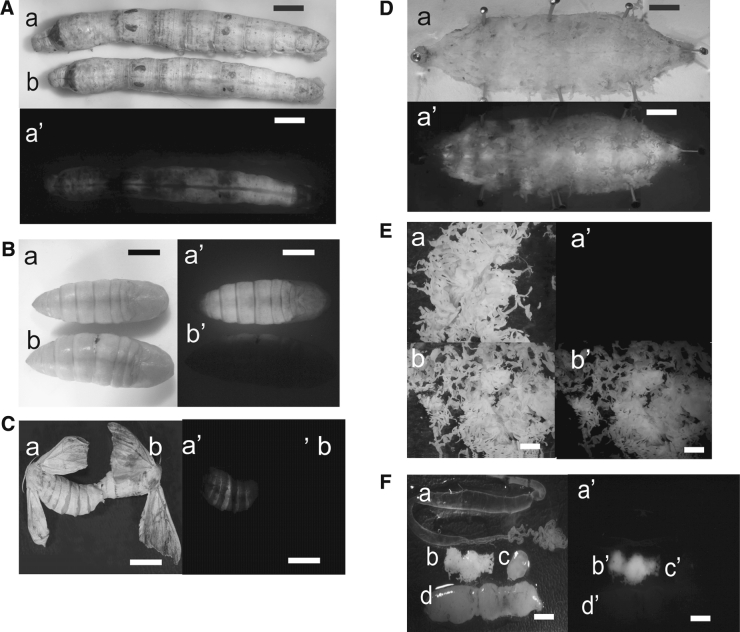
Fluorescent microscopic detection of DsRed expression in transgenic silkworm. **a** Fluorescent detection of DsRed in the transgenic day-4 fifth instar larvae. Wild type (*a*, *a′*) and transgenic silkworm (*b, b′*) were viewed under white light and 565 nm ultraviolet light (optimal for detecting RFP fluorescence). **b** Fluorescent detection of DsRed in the transgenic pupae. Transgenic (*a*, *a′*) and wild type pupae (*b*, *b'*) were viewed under white light and 565 nm ultraviolet light. **c** Fluorescent detection of DsRed in the transgenic adults. Transgenic (*a*, *a′*) and wild type adults (*b, b′*) were viewed under white light and 565 nm ultraviolet light. **d** Fluorescent detection of DsRed in dissected transgenic day-7 fifth instar larvae. Transgenic larvae was viewed under white light (*a*) and 565 nm ultraviolet light (*a′*). **e** DsRed fluorescent detection the isolated fat body from transgenic and wild-type day-7 fifth instar larvae. Fat body of wild type (*a′*) and transgenic larvae (*b, b′*) were dissected in 1× PBS and viewed under white light or 565 nm ultraviolet light. **f** Tissue-specific fluorescent detection of DsRed in the transgenic day-7 fifth instar larvae. Various tissues including the silk glands (*a′*), fat body (*b, b′*), gonads (*c, c′*) and midgut (*d, d′*) from transgenic larvae were viewed under white light and 565 nm ultraviolet light. The *scale bar* represents 0.5 cm

As shown in Fig. 3, pBacBmlp3-*DsRed*/3 × P3EGFP resulted in high levels of RFP fluorescence in transgenic progeny. RFP fluorescence could not be detected in early developmental stages until day-4 of the fifth instar of transgenic silkworm. The intensity of RFP fluorescence increased with time during larval development and could be observed clearly in the body (Fig. 3a). During the pupal stage, a bright and intense RFP fluorescence was detected in whole body, indicating the high level expression of *DsRed* in this stage (Fig. 3b). *DsRed* expression continued throughout the pupal stage but decreased in adults with no obvious differences between expression in males and females (Fig. 3c). Dissected day-7 fifth instar larvae had RFP fluorescence localized entirely within the fat body, which was distributed throughout the insect (Fig. 3d, e). No RFP fluorescence was detected in other organs including the silk gland, gonads, midgut and hemolymph (Fig. 3f). These results show that the 1,119 bp fragment from the 5′ region of *Bmlp3* has fat-body specific promoter activity.

### Spatial and temporal expression of *DsRed* directed by the *Bmlp3* promoter

**Fig. 4:**
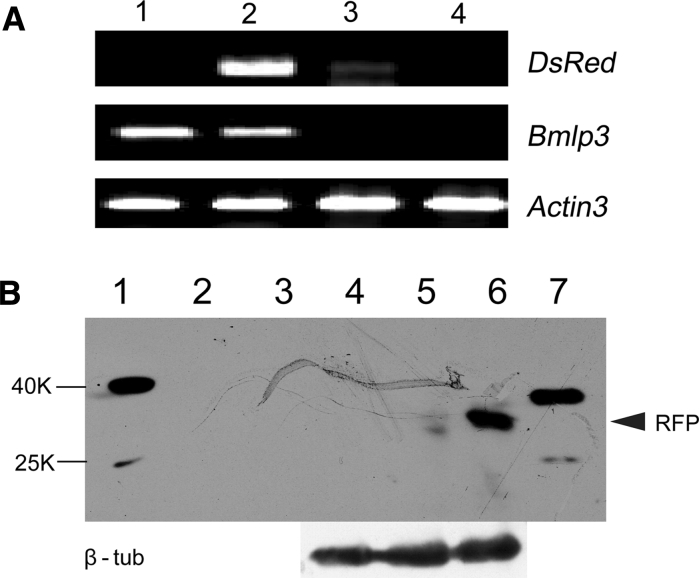
Tissue-specific expression of *DsRed* in transgenic silkworm. **a** Monitoring the level of *DsRed* mRNA in the day-7 fifth instar larvae by RT-PCR. *Lanes 1–4* are RT-PCR results using cDNA template derived from RNA isolated from the fat body of wild type (*1*), transgenic (*2*) silkworms, and the silk glands (*3*) and midgut (*4*) of transgenic silkworms. **b** Monitoring the level of *DsRed* protein in the day-7 fifth instar larvae by Western blotting. *Lanes 1–7* contain a protein marker (*1*), 10 μg of total protein from wild-type fat bodies (*2*) and hemolympth (*3*), the silk gland (*4*), midgut (*5*) and fat body (*6*) of transgenic larva and 1 μg of recombinant RFP (*7*). The *numbers on left side* of gels indicate the molecular mass (kDa). *Arrowhead* on the *right* indicates the recombinant RFP

**Fig. 5:**
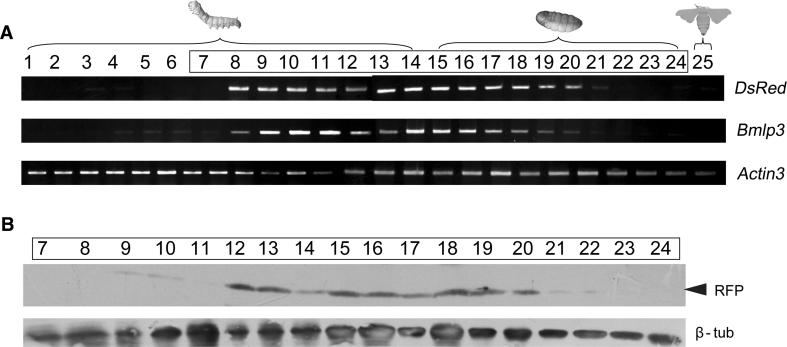
Stage-specific expression of *DsRed* in transgenic silkworm. **a** Transcriptional level of *DsRed* at different stages of transgenic silkworm by RT-PCR. *Lanes 1–25* are RT-PCR results using cDNA template derived from RNA isolated from day-1 fourth instar larvae (*1*), day-2 fourth instar larvae (*2*), day-3 fourth instar larvae (*3*), molting stage of fourth instar larvae (*4*), day-1 fifth instar larvae (*5*), day-2 fifth instar larvae (*6*), day-3 fifth instar larvae (*7*), day-4 fifth instar larvae (*8*), day-5 fifth instar larvae (*9*), day-6 fifth instar larvae (*10*), day-7 fifth instar larvae (*11*), day-1 after wandering (*12*), day-2 after wandering (*13*), day-3 after wandering (*14*), day-1 pupa (*15*), day-2 pupa (*16*), day-3 pupa (*17*), day-4 pupa (*18*), day-5 pupa (*19*), day-6 pupa (*20*), day-7 pupa (*21*), day-8 pupa (*22*), day-9 pupa (*23*), day-10 pupa (*24*), and moth (*25*). Gene-specific primers included those to detect the DsRed transgene (*DsRed*), endogenous Bmlp3 (*Bmlp3*) and Actin3 (*Bmactin3*). **b** Western blot analysis of DsRed protein expression in transgenic silkworms at different stages. *Lanes 7–24* have proteins isolated from the same stages of those *boxed lanes* in (**a**). The filter was probed using an anti-RFP antibody (RFP) then stripped and re-probed using an anti β-tubulin antibody (β-tub)

To further investigate the expression patterns of *DsRed* controlled by the *Bmlp3* promoter, mRNA was extracted from different tissues of day-7 fifth instar larvae and the fat body from different developmental stages including day-1 fourth instar larvae to adults and analyzed by RT-PCR and Western blotting. As shown in Fig. 4, strong expression of *Bmlp3*-regulated *DsRed* was detected in the fat body of transgenic silkworms, and this was consistent with the pattern of expression of the endogenous *Bmlp3* gene. Unexpectedly, low levels of *DsRed* transcripts were detected in the silk glands of transgenic silkworms, however DsRed protein was undetectable in this tissue. We speculate that regulatory elements required for the complete repression of *Bmlp3* in the silk glands might be absent from the isolated 1,119 bp DNA fragment. A developmental analysis of *DsRed* expression detected low levels of *DsRed* transcripts in day-3 fourth instar larvae through day-3 fifth instar larvae, *DsRed* transcript levels then increased with maximal expression occurring in the mid-pupal stage, and finally disappeared at day-8 pupae. The accumulation of *DsRed* mRNA follows a pattern very similar to that observed for the transcripts of the endogenous *Bmlp3* gene (Fig. 5a). Western blot analysis of DsRed protein expression confirmed the patterns of DsRed gene expression. However, the DsRed protein was only detected from day-5 fifth instar larvae and disappeared in day-9 pupae (Fig. 5b), suggesting that the DsRed protein might have been released into the hemolymph.

In addition, quantity of RFP in the fat body of transgenic silkworm is determined by densitometric measurement of the immunoblot using Quantity One software. It showed that each 10 μg fat body of transgenic silkworm contains 0.54 μg pure RFP. As we know proteins of fat body are synthesized abundantly and rapidly from late fifth instar silkworm larva to pupae, achieving about 30 % of whole body (Xiang 2005). That is to say the pure RFP synthesized by fat body account for about 1.6 % of single transgenic silkworm/pupae, suggesting the potential utility of the *Bmlp3* promoter for production of recombinant proteins in the fat body of transgenic silkworm.

## Future work

In this study, we report for the first time the isolation and characterization of a fat body-specific promoter from *Bmlp3*, a gene encoding a member of the silkworm 30K protein family. Our results demonstrated that a 1,119 bp fragment from the 5′ end of *Bmlp3* had promoter activity sufficient to direct fat body-specific expression of *DsRed* reporter gene. This promoter-containing fragment results in useful temporal and spatial patterns of gene expression and is a useful tool for functionally analyzing interesting genes in the fat body of transgenic silkworms. Next, we will focus on identifying the key regulatory elements and putative transcription factors responsible for stage- and tissue-specific expression of the *Bmlp3* promoter using single base mutagenesis and/or promoter deletion strategy, to promote our better understanding of characters of the *Bmlp3* promoter and even the synthesis and regulation of 30K proteins.

As described above, the silkworm fat body is highly active in protein synthesis. Our results demonstrated the utility of the *Bmlp3* promoter to produce recombinant proteins in the fat body of transgenic silkworm/pupae, with an obvious advantage of requiring little need for protein purification. Silkworm pupae have been a favorite food in China from ancient times (Yang et al. 2009). In addition, it can also be processed directly into animal feeds and is a valuable and inexpensive nutrient source. The recombinant proteins produced in fat body cells of the transgenic silkworm/pupae are wrapped in a coating of fat and can avoid degradation after direct cooking or feeding to animals. In our future work we will test the feasibility of using the *Bmlp3* promoter to regulate the expression of recombinant proteins such as antibiotics and animal vaccines in the fat body of transgenic silkworms.

## Electronic supplementary material

Below is the link to the electronic supplementary material.

**Fig. S1. Southern blotting and Insertion analysis of transgenic silkworms.** (A) Samples of genomic DNA extracted from transgenic and wild type silkworm were fully digested with *Hind*III (lane 1), *Xho*I (lane 2) and *Hind*III (lane 3, control), and subjected to Southern blotting analysis with EGFP probe. (B) The flanking genomic sequences obtained with insertion site TTAA on the *piggyBac* left arm and *piggyBac* right arm. The insertion site was located in the Scaffold nscaf1806 of Chromosome 20. (TIFF 317 kb)

**Table S1. Primers and probes used in amplification of the promoter region of** ***Bmlp3*** **and analysis of Southern blotting, Inverse PCR and RT-PCR of transgenic silkworms.** (DOC 37 kb)

**Table S2. Germ-line transformation experiments.** (DOC 24 kb)
