# Supplementary figures and images for: The promoter of Bmlp3 gene can direct fat body-specific expression in the transgenic silkworm, Bombyx mori
Source: Transgenic Res. 2013 Mar 30;22(5):1055–63. doi: 10.1007/s11248-013-9705-8 (PMC3781314; doi:10.1007/s11248-013-9705-8)

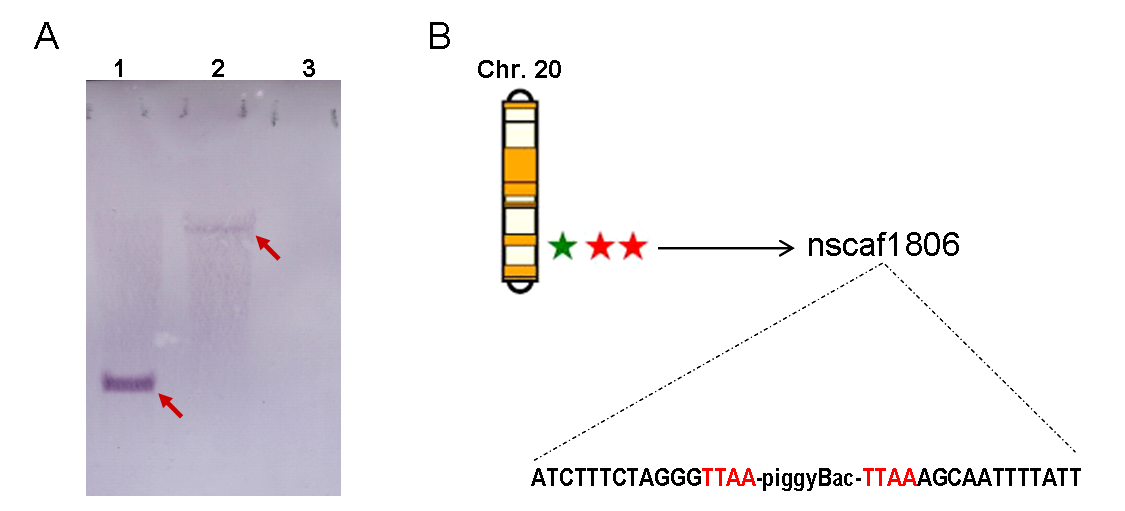

Supplement: Supplementary file 1 — Fig. S1. Southern blotting and Insertion analysis of transgenic silkworms. (A) Samples of genomic DNA extracted from transgenic and wild type silkworm were fully digested with HindIII (lane 1), XhoI (lane 2) and HindIII (lane 3, control), and subjected to Southern blotting analysis with EGFP probe. (B) The flanking genomic sequences obtained with insertion site TTAA on the piggyBac left arm and piggyBac right arm. The insertion site was located in the Scaffold nscaf1806 of Chromosome 20. (TIFF 317 kb) [file 11248_2013_9705_MOESM1_ESM.tif]
