# Supplementary material for: The promoter of Bmlp3 gene can direct fat body-specific expression in the transgenic silkworm, Bombyx mori
Source: Transgenic Res. 2013 Mar 30;22(5):1055–63. doi: 10.1007/s11248-013-9705-8 (PMC3781314; doi:10.1007/s11248-013-9705-8)
Supplement: Supplementary file 2 — Table S1. Primers and probes used in amplification of the promoter region of Bmlp3 and analysis of Southern blotting, Inverse PCR and RT-PCR of transgenic silkworms. (DOC 37 kb) [file 11248_2013_9705_MOESM2_ESM.doc]

**Table S1**

| **Primer** | **Sequence (from 5′to 3′)** | **Role** |
| --- | --- | --- |
| Bmlp3-PF1 | TCCcccgggGGATTAGTATAGTTACAACAGCT (*Sma* I) | Cloning the promoter region of *Bmlp3* gene |
| Bmlp3-PR1 | CCGctcgagCGGTCGAGTCCTGCAATATGT (*Xho* I) |  |
| Bmlp3-PF2 | GgaattcCAGTATAGTTACAACAGCTGCCCC (*EcoR* I) | Cloning the promoter region of *Bmlp3* gene |
| Bmlp3-PR2 | CGggatccCGCGTCGAGTCCTGCAATATGT (*BamH* I) |  |
| piggyBacL-F | ATCAGTGACACTTACCGCATTGACA | Verification of piggyBac insertion site in transgenic silkworm |
| piggyBacL-R | TGACGAGCTTGTTGGTGAGGATTCT |  |
| piggyBacR-F | TACGCATGATTATCTTTAACGTA |  |
| piggyBacR-R | GGGGTCCGTCAAAACAAAACATC |  |
| DsRed-F | ATGGTGCGCTCCTCCAAGAACG | Presence of *DsRed* reporter  gene in transgenic silkworm |
| DsRed-R | CTACAGGAACAGGTGGTGGCGG |  |
| Bmlp3-GF | TTGAGTTCGTTCTTGGGTAA | Presence of endogenous *Bmlp3* gene in transgenic silkworm |
| Bmlp3-GR | ACCACTGGGCTCTGAAAC |  |
| Bmactin3-F | AACACCCCGTCCTGCTCACTG | Presence of endogenous *Bmactin3* gene in transgenic silkworm |
| Bmactin3-R | GGGCGAGACGTGTGATTTCCT |  |
| EGFP probe | CCACAAGTTCAGCGTGTCCG | Validating the copy number in transgenic silkworm by southern blotting |
|  | AGTTCACCTTGATGCCGTTCTT |  |

Underlined sequences indicate the corresponding restriction enzymes.
