# Supplementary material for: The promoter of Bmlp3 gene can direct fat body-specific expression in the transgenic silkworm, Bombyx mori
Source: Transgenic Res. 2013 Mar 30;22(5):1055–63. doi: 10.1007/s11248-013-9705-8 (PMC3781314; doi:10.1007/s11248-013-9705-8)
Supplement: Supplementary file 3 — Table S2. Germ-line transformation experiments. (DOC 24 kb) [file 11248_2013_9705_MOESM3_ESM.doc]

Table S2

| Silkworm  strain | Number of injected  embryos | Number of hatched  embryos | Number of fertile  moths | Number of  backcrosses | Number of broods with EGFP-positive  larvae | Percentage of G0 transformed  moths |
| --- | --- | --- | --- | --- | --- | --- |
| *P50* | 357 | 171 | 126 | 31 | 5 | 0.16 |
